# Quantum Coding via Quasi-Cyclic Block Matrix

**DOI:** 10.3390/e25030537

**Published:** 2023-03-21

**Authors:** Yuan Li, Jin-Yang Li

**Affiliations:** 1 School of Electronic Information Engineering, Shanghai Dianji University, Shanghai 200240, China; 2 College of Information Engineering, NorthWest Agriculture and Forestry University, Xi’an 712100, China

**Keywords:** long-length quantum codes, stabilizer codes, jacket matrix, quasi-cyclic codes

## Abstract

An effective construction method for long-length quantum code has important applications in the field based on large-scale data. With the rapid development of quantum computing, how to construct this class of quantum coding has become one of the key research fields in quantum information theory. Motivated by the block jacket matrix and its circulant permutation, we proposed a construction method for quantum quasi-cyclic (QC) codes with two classical codes. This simplifies the coding process for long-length quantum error-correction code (QECC) using number decomposition. The obtained code length *N* can achieve $O(n^{2})$ if an appropriate prime number *n* is taken. Furthermore, with a suitable parameter in the construction method, the obtained codes have four cycles in their generator matrices and show good performance for low density codes.

## 1. Introduction

Quantum communication requires that environmental effects and decoherence should be reduced with reliable quantum information processing. In practice, information reconciliation in quantum key distribution (QKD) is important for the secret key rate and also affects the maximum transmission distance [1,2,3,4]. Error-correcting code generally may be applied in a quantum channel to correct errors caused by channel noise and possible interventions from eavesdroppers. To obtain an acceptable level, QECC is an essential method because of its robustness and efficiency in quantum computation [5,6,7,8,9,10]. One of the advantages of quantum computation is that its high efficiency compares favorably to classical computation; it is able to handle large-scale data that classical computation cannot. Previously, most of the construction methods for QECC focused on the generation of stabilizers, and there was little research on the long code type. Furthermore, due to some fields in quantum information becoming gradually more practical, this has prompted researchers to identify a good coding method for quantum error-correcting codes of long length. For example, encoding large-scale data has potential applications in the field of machine learning (ML) with respect to big data [11,12,13,14]. Therefore, how to efficiently express classical massive data with physics-based codes is also an important research field. To obtain a general quantum code, the question is usually converted to a problem of stabilizer generation. One typical method is to obtain the generation matrix of a quantum code based on two classical codes, which are called Calderbank–Shor–Steane (CSS) codes [6,7]. By resorting to the generalization of cyclic codes, a class of classical codes called quasi-cyclic (QC) codes can build linear codes based on algebraic structure, which has improved some fundamental minimum distances [15,16,17,18]. For instance, it can satisfy the modified version of the Gilbert–Vashamov (GV) bound [19,20,21]. It has shown good performance when applied to form quantum codes.

In the construction process for long-sequence QECCs, how to design the generators of stabilizers based on the block matrix is key. Methods from QC low density parity check (LDPC) codes have been studied by Hagiwara et al. in terms of a probabilistic method [22,23]. In 2018, Galindo et al. applied two generators to construct a quantum version with dual-containing QC [24]. Some QC short-length codes were obtained with good parameters. Not long after that, Ezerman et al., in 2019, used QC codes with large Hermitian hulls to form QECCs over fields $F_{4}$ and $F_{9}$ [25], so that a record-breaking binary QECC was obtained. Furthermore, J. Lv et al. proposed some new binary quantum codes derived from one generator quasi-cyclic codes with a stabilizer [26,27]. Recently, some researchers have considered the application of this kind of quantum code in quantum key distribution [4]. However, few researchers have paid attention to the construction of long-length quantum QC code involved with massive data. Inspired by the previous work, we presented QC code constructions of long length to generate QECCs with a family of orthogonal jacket matrices, the main property of which are that the inverse matrix can be obtained by its element-wise inverse or block-wise inverse [28,29]. Therefore, it can be realized relatively easily with a physical circuit. Furthermore, since Gallager first proposed LDPC codes in the 1960s [30], this class of classical code has shown good performance approaching the channel capacity [31,32,33,34,35]. Subsequently, its quantum versions has been investigated [22,23]. However, the achievements in this field have been explored far less than their classical counterparts. The constructed quantum codes have shown good performance for low density codes based on iterative coding in our proposed construction method.

In this paper, with the advantage of the convenient implementation of the jacket transform, we applied a quasi-cyclic method via a low-density block matrix to gain long-length quantum codes. If a prime number in the proposed construction method is taken to properly choose the jacket matrix with a basic matrix and block circular matrix combined together, then a longer length of matrix to encode classical data can be obtained.

This paper is arranged as follows: In Section 2, we present some preliminary information which is necessary for QECCs. Then, in Section 3 and Section 4, we investigate the construction of long-length quasi-cyclic quantum codes which are generated from block jacket matrices based on a basic matrix and circulant permutation matrices. Furthermore, the construction conditions with low density are also analyzed. Finally, conclusions are drawn in Section 5.

## 2. Preliminaries

Some relevant notation and basic construction methods for quantum error correction codes are first briefly reviewed below.

### 2.1. General Construction Methods of QECCs

A linear binary quantum error-correcting code [[N,k,d]] denotes that *k*-data vectors are encoded to *N*-dimensional vectors in space $P_{N}$, where *d* is the minimum distance. Consider a $2^{N}$-dimensional Hilbert space based on a complex field C

(1) $$P_{N}=C^{2^{N}}={\left(C^{2}\right)}^{⊗N},$$

where every $2^{N}$ standard basis vector in space $P_{N}$ is indexed by a classical binary vector $u\in F_{2}^{N}$ and denoted by |u〉. It is generally called a *N*-qubit state space, for which each component in the tensor product corresponds to one qubit. Similar to classical coding, a fundamental problem in quantum error correction is to generate quantum codes based on the best possible minimum distance. The general construction method for QECCs usually relies on a so-called stabilizer. A stabilizer quantum code C[[N,k,d]] can be gained from the stabilizer denoted as

(2) $$S=\prod_{i-1}^{N-k}\left(I+M_{i}^{m_{i}}\right):m_{i}\in \left\{0,1\right\},$$

where $M_{1},M_{2}\cdots M_{N-k}$ are N−k commuting generators of the stabilizer, which is the collection of orthogonal quantum state eigenvectors that refer to code words. Therefore, to form a stabilizer quantum code, N−k generators of stabilizer *S* should first be designed, which are expressed by the following generator matrix

(3) $$\begin{array}{cc} G=(G^{x} & G^{z}{)}_{(N-k)\times 2N}={\left(M_{1},M_{2},\ldots ,M_{N-k}\right)}^{T}, \end{array}$$

where $G^{x}={\left(g_{ij}^{x}\right)}_{\left(N-k\right)\times N},\;\;G^{z}={\left(g_{ij}^{z}\right)}_{\left(N-k\right)\times N}$ for 1≤i≤N−k,1≤j≤N, and x,z are the Pauli transformation. According to the property of commuting generators, the elements of its row vector satisfy that the symplectic inner product is equal to zero.

Another well known construction method for quantum codes is the CSS code which is generated by a pair of classical codes $C_{1}\left[n,k_{1},d_{1}\right]$ and $C_{2}\left[n,k_{2},d_{2}\right]$, where $k_{i}$ and $d_{i}$ are the information code length and minimum distance, respectively. This means that this class of quantum error code is a complex vector space characterized by a pair of classical binary linear codes $C_{1}$ and $C_{2}$. Here, the parity-check matrices $H_{1}$ and $H_{2}$ of the two classical codes are required to satisfy $H_{1}\cdot H_{2}^{T}=0$, i.e., every row of $H_{1}$ is orthogonal to every row of $H_{2}$. Hence, define a CSS code as a complex linear combination of vectors:

(4) $$\sum_{d^{\prime }\in C_{2}^{⊥}}|c+d^{\prime }\rangle \;\;\;\;\;\mathrm{for}\;\;\;\;c\in C$$

where $C_{2}^{⊥}$ is the dual code of the classical code $C_{2}$. According to its definition, the obtained quantum CSS code $C[\left[N,k_{1}+k_{2}-N,\min\left\{d_{1},d_{2}\right\}\right]]$ is a class of special stabilizer code, of which the generator matrix is

(5) $$\begin{array}{c} G_{c}=\left(\begin{array}{cc} H_{1} & O\\ O & H_{2} \end{array}\right). \end{array}$$

In light of the characteristics of big data processed in artificial intelligence and other fields, we shall apply the recursive relationship of a quasi-cyclic block jacket matrix to easily obtain long-length QECCs.

### 2.2. Error of Quantum Error-Correction Code and Bound

As with different quantum codes, errors are also labeled with strings in field $F_{2}$. This will occur when the states are transmitted through quantum channels. The traditional approach to error correction for quantum codes is to consider the single qubit flip error, the phase error or the phase-flip error, which can be described with three Pauli operators $\sigma_{1}$, $\sigma_{2}$ and $\sigma_{3}=\omega \sigma_{1}\sigma_{2}$, respectively. Here, ω is the primitive root of unity. Every error on *N* qubits can be denoted as $e=\sigma_{1}^{X}\sigma_{2}^{Z}$, for $X=(x_{1},x_{2},\ldots ,x_{n})$ and $Z=\left(z_{1},z_{2},\ldots ,z_{n}\right)\in F_{2}^{N}$. Reflexive stabilizer codes and CSS codes have a one-to-one correspondence by choosing a basis for the error group. In terms of the Pauli matrices, the single qubit quantum error can be described as X(a) and Z(a) for $a\in F_{2}$, whose action on |x〉∈C is given by

(6) $$X\left(a\right)|x\rangle =|x+a\rangle \;\mathrm{and}\;Z\left(a\right)|x\rangle =\omega^{ax}|x\rangle$$

Therefore, it acts on an *N*-qubit basis state $|Q\rangle =\left(q_{1},q_{2},\ldots ,q_{N}\right)$ in $F_{2}^{N}$ as follows

(7) $$\begin{array}{cc} e|Q\rangle & ={\left(-1\right)}^{Z\cdot Q}|X+Q\rangle \\ & ={\left(-1\right)}^{z_{1}\cdot q_{1}+\ldots +z_{N}\cdot q_{N}}|x_{1}+q_{1},\ldots ,x_{N}+q_{N}\rangle . \end{array}$$

Furthermore, the quantum Hamming bound of a general quantum code C[[N,k,d]] is required to satisfy the following inequality condition [36]

(8) ∑l=0t3lNl≤2N−k,

which may correct up to t=[(d−1)/2] quantum error bits.

## 3. Long-Length Coding Constructions Based on Jacket Matrix

**Figure 1:**
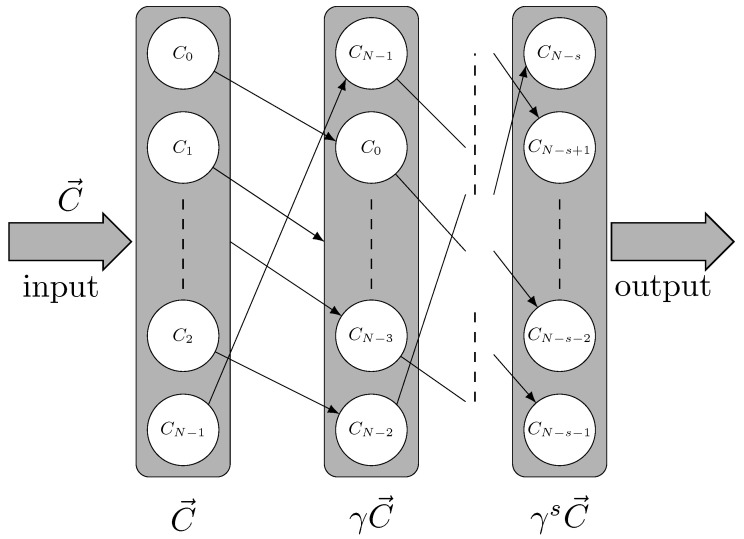
Schematic of classical cyclic code vector. Here, according to *s* times of cyclic-shift operator γ, the classical code $\vec{C}$ as an input code can generate its cyclic vectors, $\gamma \vec{C},\gamma^{2}\vec{C},\ldots ,\gamma^{s}\vec{C}$. Here, *s* is an integer number, so that the generated vectors belong to a quotient ring.

Quasi-cyclic codes are the generalization of cyclic codes, which can generate a class of linear codes of algebraic structure. Assume *C* is a classical code of length *N* over field $F_{2}$, which is closed with a cyclic-shift operator γ. This means that any codeword $\vec{C}=\left(c_{0},c_{1},\ldots ,c_{N-1}\right)\in C$ satisfies $\gamma \vec{C}=\gamma \left(c_{0},c_{1},\ldots ,c_{N-1}\right)=\left(c_{N-1},c_{0},\ldots ,c_{N-2}\right)\in C$. The circuit of the cyclic shift can be represented as in Figure 1. In this circuit, $\vec{C}$ as an input code vector can generate an output code vector with some integer *s* times of γ operation, 1≤s≤N−1.

More generally, if there is a smallest positive integer *ℓ* such that

(9) $$\begin{array}{c} \gamma^{\ell }\left(c\right)=\left(c_{N-\ell },c_{N-\ell +1},\ldots ,c_{N-1},c_{0},\ldots ,c_{N-\ell -1}\right), \end{array}$$

also belongs to *C*, the linear code *C* is called a quasi-cyclic code of index *ℓ*. Furthermore, given $C^{⊥}=\left\{\vec{v}\in F_{2}^{N}|\langle \vec{u},\vec{v}\rangle =0,\forall \vec{u}\in C\right\}$ is called its dual code. If $C\subseteq C^{⊥}$, the code *C* is self-orthogonal. In quantum theory, if any quantum code word $|c\rangle \in P_{N}$ is still a quantum code state in this space after several cyclic shifts, the quantum codes will be correspondingly called quasi-cyclic quantum codes.

In mathematical theory, a cyclic code is an ideal in the quotient ring *R*; hence, it can be gained by a single polynomial. Here, the quotient ring is defined as $R=F_{2}\left[x\right]/\langle x^{n}-1\rangle$ for a prime number *n*. It is an isomorphic $\gamma :F_{2}^{n}\to R$ between the ring formed by all n×n circulant matrices and the ring *R* formed by the polynomial $x^{n}-1$. As a consequence, the circulant matrix corresponds to the following polynomial

(10) $$\begin{array}{c} a=a_{0}+a_{1}x+\ldots +a_{n-1}x^{n-1}. \end{array}$$

Under the isomorphism γ, it has $\gamma \vec{a}=a_{n-1}+a_{0}x+\ldots +a_{n-2}x^{n-1}$, for $\vec{a}=\left(a_{0},a_{1},\ldots ,a_{n-1}\right)$$\in F_{2}^{n}$. If we define $K^{n}=I$, such as

(11) $$\begin{array}{c} K=\left(\begin{array}{cccc} 0 & 0 & \ldots & 1\\ 1 & 0 & \ldots & 0\\ 0 & 1 & \ldots & 0\\ \vdots & \vdots & \ddots & \vdots \\ 0 & 0 & \ldots & 0 \end{array}\right) \end{array}$$

is a n×n matrix representing the right cyclic shift by one position, it is readily seen that a circulant matrix *A* over $F_{2}$ can be represented in the form

(12) $$\begin{array}{c} A=a_{0}I+a_{1}K+\ldots +a_{n-1}K^{n-1}, \end{array}$$

where *I* is the n×n identity matrix. More generally, the n×n matrix $K=(k_{ij})$ may be taken as

(13) $$k_{ij}=\{\begin{array}{cc} 1 & \mathrm{if}i=(j+h)\;\mathrm{mod}\;n\\ 0 & \mathrm{otherwise}, \end{array}$$

where, *h* is an integer for 1≤h≤n−1. It is obvious that the matrix *K* can form an Abelian operator group $K=\{I=K^{0},K^{1},\ldots ,K^{n-1}\}$. The index *i* of $K^{i}\in K$ is an exponent of matrix *K*, which is called a *basic matrix*. More generally, the matrix $G_{e}$ formed by the index of the basic matrix is defined as an *exponent matrix*. On the basis of the two matrices, we also introduce a *circulant permutation matrix Q* obtained by an operation “∧” between the matrices *K* and $G_{e}$ as

(14) $$\begin{array}{cc} Q & ={\left(K^{a_{ij}}\right)}_{mn\times nn}=K\wedge G_{e}\\ & =\left(\begin{array}{cccc} K^{a_{11}} & K^{a_{12}} & \ldots & K^{a_{1n}}\\ K^{a_{21}} & K^{a_{22}} & \ldots & K^{a_{2n}}\\ \vdots & \vdots & \ddots & \vdots \\ K^{a_{m1}} & K^{a_{m2}} & \ldots & K^{a_{mn}} \end{array}\right), \end{array}$$

where $a_{ij},1\leq i\leq m,1\leq j\leq n$, is the exponent of the basic matrix *K*, and

(15) $$\begin{array}{c} G_{e}={\left(a_{ij}\right)}_{m\times n}=\left(\begin{array}{cccc} a_{11} & a_{12} & \ldots & a_{1n}\\ a_{21} & a_{22} & \ldots & a_{2n}\\ \vdots & \vdots & \ddots & \vdots \\ a_{m1} & a_{m2} & \ldots & a_{mn} \end{array}\right). \end{array}$$

It is easily seen that *Q* is essentially an array of cyclic-shift operators.

On the other hand, a class of matrix $J_{n\times n}={\left(a_{ij}\right)}_{n\times n}$ is called a *jacket matrix*, which could lead to a simple encoding algorithm [28,29], if it satisfies

(16) $$a_{ij}^{-1}=\{\begin{array}{cc} (a_{ij})/a & \mathrm{if}a_{ij}\neq 0,\\ 0 & a_{ij}=0, \end{array}$$

i.e., $J_{n\times n}^{-1}={\left(a_{ij}^{-1}\right)}^{T}/a$, where *a* is the normalized constant. It is obvious that Pauli matrices and the Hadamard matrix belong to a jacket matrix.

In addition, we also define an operation ‘$\hat{⊗}$’ between the Jacket matrix *J* and the cyclic-shift operator vector $\vec{K}=\left(I,K,\ldots ,K^{n-1}\right)$ as

(17) $$\begin{array}{c} J\hat{⊗}\vec{K}=J\hat{⊗}\left(I,K,\ldots ,K^{n-1}\right)=\left(J,JK,\ldots ,JK^{n-1}\right). \end{array}$$

Then, on the basis of the jacket matrix *J* and the circulant permutation matrix *Q*, we design a matrix *G* with length N=n(n−1) constructed by the following circuit

(18) $$\begin{array}{cc} G & =J\hat{⊗}\left(K\wedge G_{e}\right)=J\hat{⊗}Q\\ & =J\hat{⊗}\left(\begin{array}{cccc} K^{a_{11}} & K^{a_{12}} & \ldots & K^{a_{1n}}\\ K^{a_{21}} & K^{a_{22}} & \ldots & K^{a_{2n}}\\ \vdots & \vdots & \ddots & \vdots \\ K^{a_{m1}} & K^{a_{m2}} & \ldots & K^{a_{mn}} \end{array}\right)\\ & =\left(\begin{array}{cccc} JK^{a_{11}} & JK^{a_{12}} & \ldots & JK^{a_{1n}}\\ JK^{a_{21}} & JK^{a_{22}} & \ldots & JK^{a_{2n}}\\ \vdots & \vdots & \ddots & \vdots \\ JK^{a_{m1}} & JK^{a_{m2}} & \ldots & JK^{a_{mn}} \end{array}\right),\;\;\;\;\;\;\;\;\; \end{array}$$

where, $G_{e}={\left(a_{ij}\right)}_{mn}$ is the exponent matrix of the basic matrix *K* in Equation (13).

## 4. Long-Length Quantum Coding via Quasi-Cyclic Jacket Matrix

Based on the above mathematical definitions, we mainly consider how to obtain the exponent matrix $G_{e}$ and the jacket matrix *J* as follows.

### 4.1. The Construction Method of Jacket Matrix

Firstly, the construction manner of the low-parity jacket matrix *J* in Equation (18) is investigated. According to their definition, it is obvious that the Hadamard matrix and the Pauli matrices are special jacket matrices. Consider the 2-order Hadamard matrix

(19) $$H_{2}=\left(\begin{array}{cc} 1 & 1\\ 1 & -1 \end{array}\right),$$

as the smallest basic matrix. A binary $n_{1}$-size Hadamard matrix $H={\left(h_{ij}\right)}_{n_{1}\times n_{1}}$ for $n_{1}=2^{m},\;m\geq 3$, under mapping: 1→1,−1→0, may be obtained with the following recursive relation

(20) $$\begin{array}{c} H_{n_{1}}=H_{n_{1}/2}⊗H_{2}. \end{array}$$

Generally, on the basis of the tensor product, the $n_{1}=2^{m}$-order jacket matrix can be shown as

(21) $$\begin{array}{c} J_{n_{1}}=\prod_{i=1}^{m}I_{2^{m-i}}⊗J_{2}⊗I_{2^{i-1}}, \end{array}$$

where $J_{2}$ may also be chosen as Pauli matrices except for the Hadamard matrix,

(22) $$\begin{array}{c} \sigma_{0}=\left(\begin{array}{cc} 1 & 0\\ 0 & 1 \end{array}\right)\;\;\;or\;\;\;\sigma_{1}=\left(\begin{array}{cc} 0 & 1\\ 1 & 0 \end{array}\right). \end{array}$$

Correspondingly, one obtains

(23) $$\begin{array}{c} J_{n_{1}}J_{n_{1}}^{T}=\left(\prod_{i=3}^{m}I_{2^{m-i}}⊗\left(J_{2}J_{2}^{T}\right)⊗I_{2^{i-1}}\right)⊗\left(J_{2^{3}}^{T}J_{2^{3}}\right). \end{array}$$

Similarly, we also consider a jacket matrix with size $n_{2}=3^{m}$ based on the fundamental matrix $J_{3}$ as follows: Denoting a 1×1 Jacket matrix as $J_{1}=1$, the direct sum $J_{3}$ may be written as

(24) $$J_{3}=J_{1}⊕J_{2}=\left(\begin{array}{cc} 1 & 0\\ 0 & J_{2} \end{array}\right).$$

It is easy to check that

(25) $$\begin{array}{ccc} J_{3}J_{3}^{-1} & = & \left(\begin{array}{cc} 1 & O\\ O & J_{2} \end{array}\right)\left(\begin{array}{cc} 1 & O\\ O^{T} & J_{2}^{-1} \end{array}\right)=I_{3}, \end{array}$$

Then, one may obtain

(26) $$\begin{array}{c} J_{n_{2}}=J_{n_{2}/3}⊗J_{3}, \end{array}$$

for $n_{2}=3^{m},m\in \left\{1,2,\cdots \right\}$.

In terms of number theory, any finite prime number *n* may be decomposed into a Kronecker product and the direct sum of 2 and 3 resort to the following form

(27) $$\begin{array}{c} n=2^{n_{1}^{\prime }}+2^{n_{2}^{\prime }}+\cdots +2^{n_{r}^{\prime }}+3^{m_{1}^{\prime }}+\cdots +3^{m_{s}^{\prime }}, \end{array}$$

where $n_{\mu }^{\prime }$, $m_{\nu }^{\prime }$ are integers for 1≤μ≤r,1≤ν≤s. Therefore, to obtain any prime size of quantum code, we first construct generator matrices with size $2^{m}$ and $3^{m}$. In view of the recursive relation, the jacket matrix $J_{n}$ of large-scale order and its transpose matrix $J_{n}^{T}$ satisfy

(28) $$J_{n}J_{n}^{T}=\left(J_{2}^{⊗n_{1}^{\prime }}J_{2}^{⊗n_{1}^{\prime }T}\right)⊕\cdots \left(J_{2}^{⊗n_{r}^{\prime }}J_{2}^{⊗n_{1}^{\prime }T}\right)⊕\left(J_{3}^{⊗m_{1}^{\prime }}J_{3}^{⊗m_{1}^{\prime }T}\right)⊕\cdots (J_{3}^{⊗m_{s}^{\prime }}J_{3}^{⊗m_{s}^{\prime }T}).$$

It is obvious that the weight of the gained jacket matrix $J_{n_{2}}$ is equal to 1 when it involves $J_{2}$ as one of the Pauli matrices. For clarity, some decomposition methods of the jacket matrix are presented based on 2-order and 3-order *fundamental jacket matrices* $J_{2}$ (Pauli matrices or the Hadamard matrix) and $J_{3}$ in Table 1.

As can be seen from the above table, any large-scale jacket matrix of prime order can be decomposed by these two kinds of matrices.

**Example 1.**  *Take parameter n=59 that is decomposed with a Kronecker product and the direct sum of the fundamental Jacket matrices, then the concatenated matrix may be expressed as different methods*

(29) $$\begin{array}{cc} J_{59} & =J_{2}^{⊗5}⊕J_{3}^{⊗3}=J_{32}⊕J_{27}=\left(\begin{array}{cc} J_{32} & O\\ O & J_{27} \end{array}\right)\\ & =J_{7}^{⊗2}⊕J_{10}=J_{49}⊕J_{10}=\left(\begin{array}{cc} J_{49} & O\\ O & J_{10} \end{array}\right) \end{array}$$

*Therefore, the same jacket matrix may be described with different forms in the light of its decomposition methods. Apparently, both a jacket matrix J and a $K^{i}$ matrix have row weight 1 with any positive integer 1≤i≤l, so that the row weight of G is exactly n−1, for a given prime n>2, where the block length of the constructed code is N=n(n−1).*

### 4.2. The Construction Method of the Exponent Matrix

Next, based on the obtained basic matrix *K*, to generate the stabilizer with two circulant permutation matrices, we consider further the construction algorithm of the exponent matrix $G_{e}$ in Equation (18) as follows:

Assume an Abelian group $Z_{n}=\left\{0,1,\ldots ,n-1\right\}$ for a prime number n>2, and its subset $Z_{n}^{*}$ obtained by non-zero elements, i.e., $Z_{n}^{*}=Z_{n}/\left\{0\right\}$. As *n* is a prime number, the size of $Z_{n}^{*}$ is even. Hence, the set $Z_{n}^{*}$ can be divided on average into two subsets

$Z_{l_{1}}^{*}=\left\{2k+1,0\leq k\leq l-1\right\}$ and $Z_{l_{2}}^{*}=\left\{2k,1\leq k\leq l\right\}$ of no common element. As can be seen from the group, it is clear that any $a\in Z_{l_{1}}^{*},b\in Z_{l_{2}}^{*},a\neq b$ and the orders of both the subsets are half of n−1, i.e., $|Z_{l_{1}}^{*}\left|=\right|Z_{l_{2}}^{*}|=\left(n-1\right)/2=l$. Denote all the elements in $Z_{n_{i}}^{*}$ to be arranged as the first row vector ${\vec{e}}_{ij}$ of an obtained matrix $E_{i}$, where i=1,2 is the *i*th subset and j=1,2,…,andl is the *j*th permutation, so the number of the maximum sort order is *l*.

To the first vector ${\vec{e}}_{11}=\left(e_{11},e_{12},\ldots ,e_{1l}\right)$, we take the described anticlockwise (or clockwise) cyclic shift *K* in (13), i.e., ${\vec{e}}_{11}K=\left(e_{1l},e_{11},\ldots ,e_{1,l-1}\right)$. After l−1 times of operation *K* to vector ${\vec{e}}_{11}$, *l* vectors may be generated as the matrix $E_{1}$. With a similar rule, we take the sequential arrangement ${\vec{e}}_{21}=\left(e_{21},e_{22},\ldots ,e_{2l}\right)$ as the first vector of another matrix $E_{2}$. It is required that ${\vec{e}}_{21}$ is a different sequence from ${\vec{e}}_{11}$. Subsequently, the elements in the two subsets can be constructed as two exponent block matrices $E_{1}$ and $E_{2}$. The exponent matrix is generated by combining them. Namely, define an operator ‘⊙’ for combining the two sub-matrices as

(30) $$\begin{array}{c} G_{e}=E_{1}⊙E_{2}=\left(E_{1}|E_{2}\right)=\left(\begin{array}{cc} {\vec{e}}_{11} & {\vec{e}}_{21}\\ {\vec{e}}_{12} & {\vec{e}}_{22}\\ \vdots & \vdots \\ {\vec{e}}_{1l} & {\vec{e}}_{2l} \end{array}\right). \end{array}$$

Here, $E_{i}$ is an exponent sub-matrix for obtaining the exponent matrix $G_{e}$, and ${\vec{e}}_{i}$ is the row vector of the exponent sub-matrix $E_{i}$, i=1,2. On the basis of the same rule, two exponent matrices $G_{e_{1}}$ and $G_{e_{2}}$ may be gained from the elements in $Z_{l_{1}}^{*}$ and $Z_{l_{2}}^{*}$, respectively. After constructing the exponent matrix $G_{e}$ and the basic matrix *K*, the circulant permutation *Q* may be obtained. By combining it with the *n*-order jacket matrix, the nl×n(n−1) generator matrix *G* is finally obtained.

Furthermore, if a greater code rate is required, we also apply the described method again to add the rows. This purpose will be achieved to make the first vector begin with a different cyclic-shifting permutation vector with opposite direction, i.e., clockwise (or anticlockwise). As a result, it will obtain a n(n−1)×n(n−1) generator matrix *G* as

(31) $$\begin{array}{c} G_{e}=\left(\begin{array}{cc} E_{1} & E_{2}\\ E_{1}^{\prime } & E_{2}^{\prime } \end{array}\right), \end{array}$$

where $E_{1}^{\prime }$ and $E_{2}^{\prime }$ are newly obtained matrices with the similar method. It is obvious that the row weight of the obtained matrix is equal to n−1. In fact, if we wish to further reduce the weight of the row matrix, a zero block matrix can be used during the design process. If we denote “$\tau_{0}$” as the exponent of the n×n zero matrix, just taking “$\tau_{0}$” instead of 1≤t≤l−1 elements in $Z_{l_{1}}^{*}$ and $Z_{l_{2}}^{*}$, the row weight will reduce 2t in the obtained matrix.

**Figure 2:**
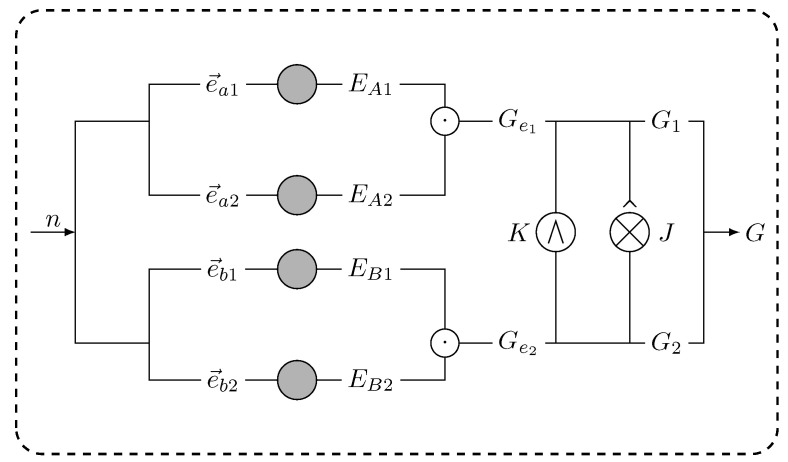
The circuit of the constructing generator matrix *G*. After taking the proper prime number *n*, two exponent matrices $G_{e_{1}}$ and $G_{e_{2}}$ may be obtained by resort to the operator ‘⊙’ on matrices $E_{A_{i}}$ and $E_{B_{j}}$ using a cyclic shift to vectors ${\vec{e}}_{a_{i}}$ and ${\vec{e}}_{b_{j}}$, respectively. Correspondingly, the quasi-cyclic matrix *H* of the quantum codes can be obtained based on the two classical quasi-cyclic matrices $G_{1}$ and $G_{2}$, which are yielded by the same jacket matrix and two circulant permutation matrices by resort to the operation ‘$\hat{⊗}$’.

The whole physical circuit of the construction process can be described as in Figure 2. In the process, a prime number *n* will be divided into two branches. According to the designed approach, two matrices $G_{e_{1}}$ and $G_{e_{2}}$ that generated similarly to $G_{e}$ will be obtained by operating a cycle-shift operation γ on two groups of vectors ${\vec{e}}_{a_{i}}$ and ${\vec{e}}_{b_{j}}$ (i,j=1,2). Finally, we take the obtained matrix $G_{i}$ (similar matrix *G* in Equation (18)) as the parity-check matrix $H_{i}$ of a classical QC code by resort to the algorithm.

However, two conditions of coding construction should be satisfied for the generated matrix *G*. On the one hand, any two rows in *G* should be orthogonal, i.e., it is self-orthogonal. In fact, according to the proposed construction algorithm, take a cyclic-shift operator σ in the no-unit operator group K/{I}, where the group K is formed by *K* introduced before. Without losing generality, denote ${\vec{v}}_{\sigma }=\left(\sigma^{i_{1}},\sigma^{i_{2}},\ldots ,\sigma^{i_{l}}\right)$ and ${\vec{v^{\prime }}}_{\sigma }=\left(\sigma^{i_{1}^{\prime }},\sigma^{i_{2}^{\prime }},\ldots ,\sigma^{i_{l}^{\prime }}\right)$ two row operator vectors in the circulant permutation matrix *Q*, $1\leq i,i^{\prime }\leq l=\left(n-1\right)/2$. Namely, exponent vectors ${\vec{e}}_{i}=\left(i_{1},i_{2},\ldots ,i_{l}\right)$ and ${\vec{e^{\prime }}}_{i}=\left(i_{1}^{\prime },i_{2}^{\prime },\ldots ,i_{l}^{\prime }\right)$ are both in the same matrix $E_{1}$ or $E_{2}$. It is obvious that, if any binary row vector $\vec{c}$ in the jacket matrix *J* is both operated by ${\vec{v}}_{\sigma }$ and ${\vec{v^{\prime }}}_{\sigma }$ with operation $\hat{⊗}$, their inner product is equal to 0 or 1, i.e., $\left(\vec{c}\hat{⊗}{\vec{v}}_{\sigma }\right)\cdot {\left(\vec{c}\hat{⊗}{\vec{v^{\prime }}}_{\sigma }\right)}^{T}=0$ or 1. Therefore, if any two row vectors $\vec{x}$ and $\vec{y}$ in matrix *J* are operated by the concatenated vector $\vec{v}=\left({\vec{v}}_{\sigma }|{\vec{v^{\prime }}}_{\sigma }\right)$ with size 2l and another vector $\gamma \vec{v}$, their inner product will always be 0 for mode 2 with one-to-one mapping, i.e., $\left(\vec{x}\hat{⊗}\vec{v}\right)\cdot {\left(\vec{x}\hat{⊗}\gamma \vec{v}\right)}^{T}=0$; here, γ is a cyclic-shift operator. This means that *G* is a self-orthogonal matrix.

On the other hand, it should also be proved that any codewords $c_{1}$ from $C_{2}^{⊥}$ are orthogonal to all codewords $c_{2}$ from $C_{1}^{⊥}$, i.e., $G_{1}\cdot G_{2}^{⊥}=0$. In fact, for the basic matrix *K*, assume its cyclic-shift time of operating γ on it in $Z_{l_{1}}^{*}$ and $Z_{l_{2}}^{*}$, respectively. According to the described algorithm, the cyclic-shift steps in the two sets are both 2, i.e., with the same operation $\gamma^{2}$. Therefore, any two matrices $K_{1}$ and $K_{2}$ of which exponents from $Z_{l_{1}}^{*}$ and $Z_{l_{2}}^{*}$, can be described as forms like Equation (12), i.e.,

(32) $$\begin{array}{c} K_{1}=a_{1}K+a_{3}K^{3}+\ldots +a_{n-1}K^{n-1},\\ K_{2}=a_{2}K^{2}+a_{4}K^{4}+\ldots +a_{n}K^{n}.\;\;\;\;\; \end{array}$$

It is obvious that $K_{1}$ and $K_{2}$ are linearly independent, i.e., the two generated Abelian spaces are orthogonal.

As a result, if we take $G_{e_{1}}$ and $G_{e_{2}}$ as the exponent matrices constructed by Equation (18), we can obtain two *N*-length matrices $G_{1}$ and $G_{2}$. Then, the $N-k_{i}$ rows of the two matrices are randomly chosen as the parity-check matrix $H_{i}$ of classical code $C_{i}\left[N,k_{i},d_{i}\right]$, (i=1,2), so a generator matrix $G_{c}$ of a QC quantum code $C[\left[N,k_{1}+k_{2}-N,\min\left\{d_{1},d_{2}\right\}\right]]$ of the form of Equation (5) is obtained.

**Example 2.**  Let prime number $n=11,Z_{1}^{*}=\left\{1,2,\cdots ,10\right\},$ so l=(n−1)/2=5, and sets $Z_{l_{1}}^{*}=\left\{1,3,5,7,9\right\}$, $Z_{l_{2}}^{*}=\left\{2,4,6,8,10\right\}$. Take ${\vec{e}}_{11}=\left(1,3,5,7,9\right)$ and ${\vec{e}}_{21}=\left(3,5,7,9,1\right)$ as the first vectors to construct the sub-matrices $E_{A1}$ and $E_{A2}$, respectively, so that $G_{e1}$ is obtained by combining the two sub-matrices. Similarly, also take ${\vec{e}}_{22}=\left(2,4,6,8,10\right)$ and ${\vec{e}}_{22}=\left(4,6,8,10,2\right)$ as the first vectors, then $G_{e2}$ will be constructed by coupling $E_{B1}$ and $E_{B2}$. Correspondingly, in terms of the basic matrix K with parameter h=1 in Equation (13), the exponent matrices $G_{e_{1}}$ and $G_{e_{2}}$ may be generated as

(33) $$\begin{array}{cc} G_{e_{1}} & =\left(E_{A1}⊙E_{A2}\right)=(E_{A1}|E_{A2})\\ & =\left(\begin{array}{cccccccccc} 1 & 3 & 5 & 7 & 9 & 3 & 5 & 7 & 9 & 1\\ 9 & 1 & 3 & 5 & 7 & 1 & 3 & 5 & 7 & 9\\ 7 & 9 & 1 & 3 & 5 & 9 & 1 & 3 & 5 & 7\\ 5 & 7 & 9 & 1 & 3 & 7 & 9 & 1 & 3 & 5\\ 3 & 5 & 7 & 9 & 1 & 5 & 7 & 9 & 1 & 3 \end{array}\right). \end{array}$$

*Similarly, by making use of a similar method, matrix $G_{e_{2}}$ may be obtained as follows:*

(34) $$\begin{array}{cc} G_{e_{2}} & =E_{B1}⊙E_{B2}=(E_{B1}|E_{B2})\\ & =\left(\begin{array}{cccccccccc} 2 & 4 & 6 & 8 & 10 & 4 & 6 & 8 & 10 & 2\\ 10 & 2 & 4 & 6 & 8 & 2 & 4 & 6 & 8 & 10\\ 8 & 10 & 2 & 4 & 6 & 10 & 2 & 4 & 6 & 8\\ 6 & 8 & 10 & 2 & 4 & 8 & 10 & 2 & 4 & 6\\ 4 & 6 & 8 & 10 & 2 & 6 & 8 & 10 & 2 & 4 \end{array}\right). \end{array}$$

*We also take the 11 by 11 basic-matrix $J_{11}=J_{6}⊕J_{5}$. Then, the matrices $G_{1}$ and $G_{2}$ with size 55×110 are obtained by combining the two exponent matrices and the basic-matrix. It is obvious that the obtained matrix can encode 55 information bits at most. To increase the coding rate k/N, we can also add the rows $E_{A1}^{\prime }$ and $E_{A2}^{\prime }$ ($E_{B1}^{\prime }$ and $E_{B2}^{\prime }$) of the exponent matrix in terms of the algorithm described before, such as*

(35) $$\begin{array}{c} \left(E_{A1}^{\prime }|E_{A2}^{\prime }\right)=\left(\begin{array}{cccccccccc} 5 & 7 & 9 & 1 & 3 & 3 & 5 & 7 & 9 & 1\\ 7 & 9 & 1 & 3 & 5 & 5 & 7 & 9 & 1 & 3\\ 9 & 1 & 3 & 5 & 7 & 7 & 9 & 1 & 3 & 5\\ 1 & 3 & 5 & 7 & 9 & 9 & 1 & 3 & 5 & 7\\ 3 & 5 & 7 & 9 & 1 & 1 & 3 & 5 & 7 & 9 \end{array}\right) \end{array}$$

*and*

(36) $$\left(E_{B1}^{\prime }|E_{B2}^{\prime }\right)=\left(\begin{array}{cccccccccc} 6 & 8 & 10 & 2 & 4 & 4 & 6 & 8 & 10 & 2\\ 8 & 10 & 2 & 4 & 6 & 6 & 8 & 10 & 2 & 4\\ 10 & 2 & 4 & 6 & 8 & 8 & 10 & 2 & 4 & 6\\ 2 & 4 & 6 & 8 & 10 & 10 & 2 & 4 & 6 & 8\\ 4 & 6 & 8 & 10 & 2 & 2 & 4 & 6 & 8 & 10 \end{array}\right).$$

*As the result, the exponent matrices $G_{e_{1}}$ and $G_{e_{2}}$ can also be shown as the following 110×110 matrix*

(37) $$\begin{array}{c} G_{e_{1}}=\left(\begin{array}{cc} E_{A1} & E_{A2}\\ E_{A1}^{\prime } & E_{A2}^{\prime } \end{array}\right)\;\mathrm{and}\;G_{e_{2}}=\left(\begin{array}{cc} E_{B1} & E_{B2}\\ E_{B1}^{\prime } & E_{B2}^{\prime } \end{array}\right). \end{array}$$

*Therefore, the two matrices $G_{1}$ and $G_{2}$ are used to form the generator matrix $G_{c}$ in Equation (5).*

For example, if we wish to obtain a quantum code with a coding rate of 0.4, by applying the proposed approach, the two classical codes $C_{1}\left[110,80,12\right]$ and $C_{2}\left[110,74,14\right]$ can be constructed, respectively. Therefore, according to the construction method of CSS-type, a quantum code C[110,44,12] is obtained. With greatly increasing code length of QC codes, some methods of computing the minimum distance are required [37,38].

**Figure 3:**
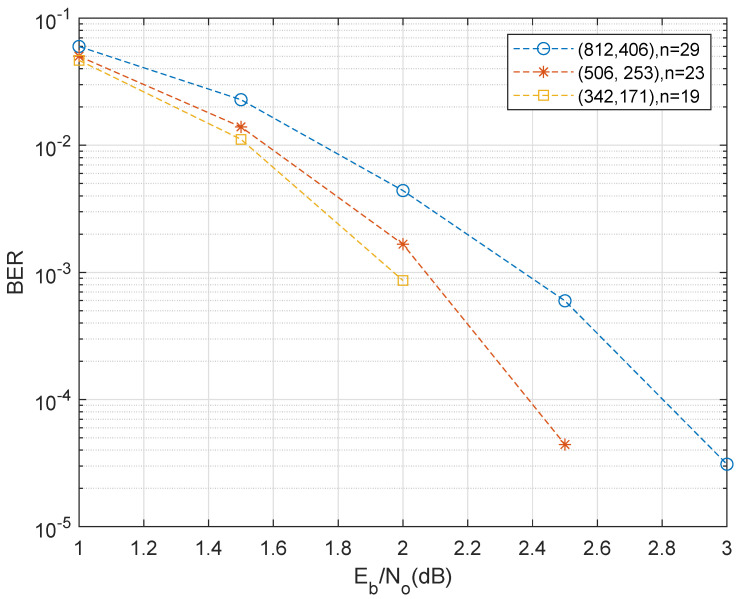
BER performance comparison of code rate R=0.5 classical QC code with N=812,N=506 and N=342. To achieve a greater length of cycle, the decomposition numbers of prime *n* more than 4 (to avoid 4−cycles) are used in our construction method. Assuming the input is a Gaussian white noise channel model, we take the prime numbers n=29,23,19 and the maximum iterations of operator γ as n−1 to construct the jacket matrixes; then the code lengths are 812,506,342, respectively.

In the following, we use different parameters to analysis the bit error ratio (BER) of the presented QC codes with a jacket matrix of 0.5 code rate. We first consider the coding performance of one of the pair of classical codes $C_{1}$ and $C_{2}$ in Figure 3. Here, if different prime numbers are taken n=29,23,19 in the construction method, their corresponding code lengths are N=n(n−1)=812,506,342, respectively. With a 0.5 coding rate, we compare their corresponding QC codes. In the signal-to-noise ratio (SNR) range, it is shown that the two shorter lengths of the taken QC quantum codes show better performance from 0.2 to 0.4 dB than the longer one. However, owing to the close cycles and their bit-flip error correction capability, a greater length of code shows its superiority with increasing length.

**Figure 4:**
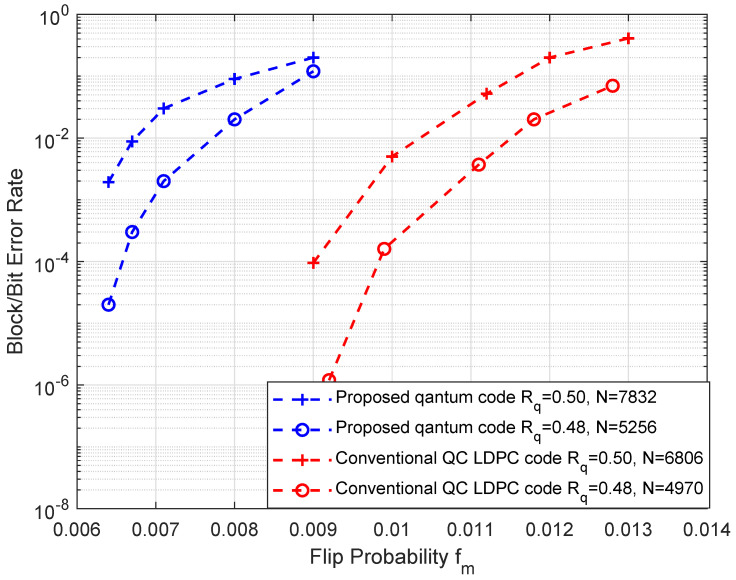
Comparison of decoding error rate of the proposed QC quantum codes (bule) and conventional QC LDPC CSS codes (red). Here, the proposed QC quantum codes with lengths N=7832 and N=5256 are generated by n=89 and n=73 for n−1 times of iterations on a binary symmetric channel, and the conventional QC LDPC codes are obtained with similar lengths with rates $R_{q}$ = 0.50, 0.48, respectively. Based on the equal error rate of one of the classical pair codes $C_{1}$ and $C_{2}$, the bit error rate of the marginal flip probability $f_{m}$ of *X* and *Z* errors is shown.

Furthermore, we consider the probability of three classes of Pauli errors in the communication channel. Assuming the channel is viewed as a possibly more realistic depolarizing channel, it can independently generate a list of *X* bit-flip errors, *Z* phase-flip errors and *Y* errors which are a combination of bit and phase flip with equal probability f/3 for a total probability *f*. Hence, the marginal flip probability distributed identically is $f_{m}=2f/3$. Owing to the construction methods of all CSS-type quantum codes obtained by the two classical codes $C_{1}$ and $C_{2}$, the *X*-and-*Z* errors may be separately decoded and corrected with a standard classical correction algorithm [39]. In Figure 4, we compare the proposed QC quantum codes with the conventional quantum error-correction codes proposed in [22]. Here, we take, respectively, the quantum coding rate of these codes as 0.50 and 0.48. The proposed codes of lengths N=7832 and N=5256 are generated by jacket matrices with parameters n=89 and n=73, respectively. According to the construction method in [22], two codes with similar lengths N=6806 and N=4970 are compared. Because the pair of classical codes $C_{1}$ and $C_{2}$ are isomorphic, the average decoding performances are similar in simulation. Therefore, we just focus on analysis of the single classical code in Figure 4. According to the presented coding method, the obtained generator matrix has sparse properties in terms of the character of the jacket block matrix; hence, a larger minimum distance for the generator matrix can be obtained. As a result, the bit error rate of our gained codes is higher than for the conventional codes, although the floor of no error for both codes is down to about $10^{-6}$. However, with increasing $f_{m}$, their BERs tend to the same level. Therefore, the proposed QC quantum codes show better performance for the coding of large-scale data.

Assume $J_{\iota }$ is a ι×ι jacket in Equation (23) constructed with Pauli matrices $\sigma_{0}$ and $\sigma_{1}$ (not including the Hadamard matrix) in the described algorithm. To analysis the computation complexity, we first consider the 0 and 1 densities in the binary matrix denoted by

(38) $$\begin{array}{c} d_{0}=\frac{\iota_{1}}{\iota_{0}+\iota_{1}}, \end{array}$$

where $\iota_{0}$ and $\iota_{1}$ are the 0 and 1 numbers respectively. Therefore, the density $d_{0}$ of the jacket matrix equals 1/ι and becomes lower with increasing length ι. According to the described algorithm, there are two operators, i.e., the Kronecker-product operator ‘⊗’ and the direct-sum operator ‘⊕’, involved. It is obvious that the calculation complexity of the direct sum is O(1) that is less than the Kronecker-product operator’s O(ι).

Therefore, the low complexity of the coding method should obey the following rules:

(1) the length of the taken matrices between the Kronecker-product operator should be larger;
(2) to obtain the long QC quantum code, the number in the decomposition method should include more direct-sum operators than Kronecker-product operators. As a result, the constructed quantum codes show good performance for low density code.

In addition, we consider the low-density properties of the constructed matrix. After first being proposed by Gallager, LDPC codes were rediscovered several decades later [31,32]. Classical LDPC codes have good performance, which approach the channel capacity in the limit of large block size. There has been considerable interest in finding quantum versions of these codes [33,34,35]. However, quantum LDPC codes are far less studied than their classical counterparts. In spite of its good performance, one main obstacle is how to obtain a highly efficient algorithm for iterative coding. In our paper, we have tentatively presented a method of constructing quantum LDPC codes and the results further enrich the theoretical achievements in this field. To ensure that the Tanner graph of the LDPC codes is free of 4-cycles, the girth may be at least 6 because the cycle of short length will reduce the performance of the LDPC codes. To meet the requirement, we just take the parameter *n* of the basic jacket matrix to be no less than 7, i.e., l≥3, in our construction algorithm.

In classical codes, a bipartite Tanner graph consists of check nodes and variable nodes [40]. The variable and check node degrees are denoted as η and ρ, respectively, which correspond to the row and column minimum distances of the sparse parity-check matrix *H*. Assume the length of the shortest cycle on the graph, and that the number of independent iterations are referred to as the girth *g* and *T*, respectively [41,42]. Correspondingly, a useful iteration gain is generally bounded by

(39) $$\begin{array}{c} T<g/4\leq T+1. \end{array}$$

**Figure 5:**
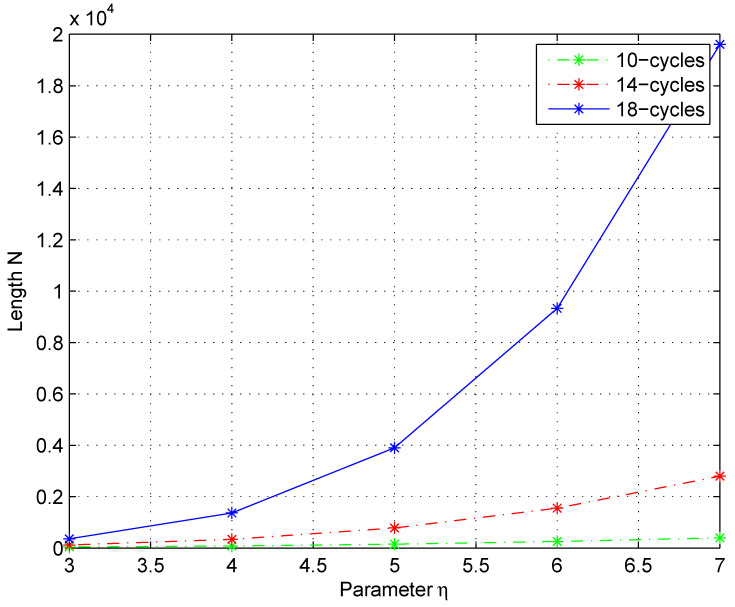
The performance of required code length *N* with different parameters. Here, to reflect the relation of several parameters, it takes x=2,3,4 so that 10-cycles, 14-cycles and 18-cycles are obtained with hypothesis η=ρ. To satisfy the bound, the prime number *n* in the proposed method should also meet the condition of length *N* and cycles.

Obviously, the gained block length should be sufficient while the girth is given, so that the bound satisfies [42]

(40) $$\begin{array}{c} N\geq 1+\sum_{i=2}^{x+1}\eta {\left(\eta -1\right)}^{i-2}{\left(\rho -1\right)}^{i-1} \end{array}$$

for a specific girth g=4x+2, where *x* is an integer. According to the bound (40), the relation of the code length *N* and the weights η and ρ is shown in Figure 5. Here, as *x* is taken as 2, 3 and 4 with hypothesis η=ρ, then the cycle-10, cycle-14 and cycle-18 are gained, respectively.

In this figure, it is shown that the length increases exponentially with the cycle length and its minimum weight η. Provided that the required cycle is determined, i.e., the cycle is bigger than a 4-cycle, for balancing the weights η,ρ and the code length *N*, it is necessary to consider the fundamental construction of the jacket matrix. In terms of the characters of the proposed method, the minimum distance of the obtained codes will grow linearly with increase in the parameter *n*. Thereby, we can apply the taken prime number to generate jacket matrices to adjust the relation between η and *N*. The main rule for reducing the density and increasing the cycle of the parity check matrix *H* is that *n* in the decomposition method should be larger. For example, if we decompose the length n=156 of the QC code as $J_{156}=J_{13}⊗J_{3}⊗J_{2}^{⊗2}$ with the most basic method, the cycles will be smaller. However, if we take another decomposition method $J_{156}=J_{7}⊗J_{11}⊕J_{43}⊕J_{23}⊕J_{13}$, the cycle length is obviously increased, and, hence, the obtained quantum codes have the advantage of simple implementation of an iterative decoder and low-complexity encoding.

According to information coding theory, the sparse parity-check matrix *H* of code [N,k] for decoding can be gained from the generator matrix. Based on the character of our constructed matrix with prime number *n*, it is easy to check that the variable and check node degrees in *H* or the corresponding bipartite Tanner graph at most are η=n−1 and ρ=[k/n]+1, where [·] is an integral function. For Example 2, the row and column weights of the parity-check matrix for constructed codes [110,80] are η=9 and ρ=8, respectively. It is obvious that the bound can be met with its 6-cycle for x=1.

Furthermore, according to the family of proposed codes from the circulant permutation matrix in Equation (14), the obtained quantum QC code of dimension O(N) contains its upper bound on the minimum distance O(N/logN) as the code length N→∞. This means that, the adopted parameter *n* in the proposed coding method should correspondingly approach

(41) $$\begin{array}{c} n\approx \sqrt{\sum_{i=2}^{x+1}\eta {\left(\eta -1\right)}^{2(i-2)}},\;\;\;1<x\in Z \end{array}$$

with the long-code length, where Z is an integer set.

In practical applications, quantum codes with long length may potentially be used in some future quantum information fields involved in large-scale data, such as quantum machine learning, which uses quantum computers and aims to enhance the power of machine learning. However, some major obstacles still limit the use of quantum hardware for practical applications of machine learning. One of the bottlenecks is that the data has to be encoded into the quantum computer in an efficient manner to generate its useful quantum kernel. Although some encoding methods have been investigated [43,44,45], the features are limited by the number of qubits [46]. Additionally, the inherent noise of quantum computers also influences the quality of the experimental results. Generally, rapid processing of the large datasets is vital because the operation timescale relies linearly on the size of the dataset. Therefore, to provide a useful kernel for machine learning, a good coding manner with long code length can efficiently load a high-dimensional feature vector into a quantum computer. As a consequence, the proposed coding method in this paper provides a tentative coding scheme for ML based on large-scale data.

## 5. Conclusions

The coding method for massive data has important applications in the field of information transmission and big-data processing. With the rapid development of quantum computing, this class of coding construction has become one of the research fields in quantum information. In this paper, based on a class of circulant permutation matrices, we presented quantum quasi-cyclic CSS codes in terms of two long-length classical codes based on block matrices. Motivated by the advantages of convenient and low-complexity implementation of an iterative decoder for proposed code, a family of jacket block matrices were applied to quantum coding construction. The presented method can efficiently construct long-length quantum QC codes based on the proposed method of an iterative coding process. If the parameter is selected appropriately, the obtained quantum codes are number 4-cycles in their generators and have good performance for LDPC codes.

Furthermore, we also analysed the marginal flip probability $f_{m}$ with respect to our proposed codes; that a good performance was obtained for the bit error rate is shown in Section 4. Therefore, the obtained QC quantum codes that benefit from their semi-parallel architectures can potentially be applied to the future quantum information field in relation to massive data.

## Figures and Tables

**Table 1:** The construction of jacket matrices.

Jacket Matrix	Decompositions of Prime Number
$J_{5}$	$J_{2}⊕J_{3}$
$J_{7}$	$J_{2}^{⊗2}⊕J_{3}$
$J_{29}$	$J_{3}^{⊗3}⊕J_{2}$ = $J_{2}^{⊗4}⊕J_{13}$
$J_{31}$	$J_{3}^{⊗3}⊕J_{2}^{⊗2}$ = $J_{2}^{⊗3}⊕J_{23}$
$J_{37}$	$J_{2}^{⊗5}⊕J_{5}$ = $J_{2}^{⊗4}⊕J_{3}⊗J_{7}$

## Data Availability

The data used to support the findings of this study are included within the article.
